# Why do seizures occur when they do? Situations perceived to be associated with increased or decreased seizure likelihood in people with epilepsy and intellectual disability

**DOI:** 10.1016/j.yebeh.2014.08.016

**Published:** 2014-10

**Authors:** Josephine L. Illingworth, Peter Watson, Howard Ring

**Affiliations:** a Department of Psychiatry, University of Cambridge, Douglas House, 18d Trumpington Road, Cambridge CB2 8AH, UK; b Medical Research Council Cognition and Brain Sciences Unit, 15 Chaucer Road, Cambridge CB2 7EF, UK; c Cambridgeshire & Peterborough NHS Foundation Trust, Cambridge, UK; d NIHR Collaboration for Leadership in Applied Health Research and Care (CLAHRC) East of England, Cambridge, UK

**Keywords:** Intellectual disability, Epilepsy, Seizure precipitant, Inhibiting factor, Genetic syndrome, Survey

## Abstract

Seizure precipitants are commonly reported in the general population of people with epilepsy. However, there has been little research in this area in people with epilepsy and intellectual disability (ID). We conducted a survey of the situations associated with increased or decreased seizure likelihood in this population. The aim of the research was to identify situations of increased seizure likelihood (SISLs) and situations of decreased seizure likelihood (SDSLs) reported by carers of people with an ID and epilepsy. Three study groups were investigated: two groups comprising individuals with ID associated with a specific genetic diagnosis – Rett syndrome or fragile X syndrome – and one group consisting of individuals with a range of other etiologies. Responses relating to 100 people were received: 44 relating to people with Rett syndrome, 25 to people with fragile X syndrome, and 31 to people whose ID had some other etiologies. Ninety-eight percent of the respondents reported at least one SISL, and 60% reported at least one SDSL. Having more seizure types and greater seizure frequency were associated with a higher number of SISLs reported. The most commonly reported SISLs and SDSLs for each of the three groups are presented. The most common SISL overall was illness, which was reported as an SISL by 71% of the respondents. There was less consensus with regard to SDSLs. These findings provide a greater understanding of when seizures occur in those with ID and epilepsy, with possible implications for adjunctive behavioral management of seizures in those with treatment-refractory epilepsy.

## Highlights

• A survey of seizure occurrence in people with epilepsy and intellectual disability
• Carers reported the situations they associated with increased and decreased seizure likelihood.
• The study included carers of those with Rett syndrome, Fragile X syndrome, and other etiologies.
• 98% of the respondents reported at least one situation of increased seizure likelihood.
• The most commonly reported situation of increased seizure likelihood was illness.

## 1 Introduction

In people with established epilepsy, the important question – ‘why do seizures occur when they do?’ – remains largely unanswered. There have been several surveys of people with epilepsy in the general population that have indicated that a substantial proportion identify environmental, physical, or emotional factors that are associated with either increased or decreased likelihood of seizure occurrence [1–10]. Stress is the most commonly reported seizure precipitant in a number of surveys of people with epilepsy or their carers [2,5,8,10,11], with tiredness and sleep deprivation also commonly mentioned [4,5,7,8,10,12]. Both sleep deprivation and stress have been found to independently predict seizure occurrence in a recent prospective study [13] (though this was not replicated [14]). Other research in the general population of people with epilepsy has found a number of other variables, such as menstrual status [15] and time of day [16], to be associated with altered likelihood of seizure occurrence, in addition to precipitants for the so-called ‘reflex epilepsies’, such as photic stimulation [17]. Investigating potential relationships between physical, emotional, and environmental factors and the occurrence of seizures is important as it may contribute to a better understanding of mechanisms underlying the generation of seizures and could potentially suggest behavioral management strategies for reducing seizure occurrence.

Despite this large quantity of research focusing on people with epilepsy within the general population, there has been little research on this topic in people with intellectual (learning) disabilities (ID). Addressing the lack of research on this topic in people with ID is warranted for a number of reasons. First, those with ID have particularly high rates of epilepsy, and many continue to have seizures despite their antiepileptic drug (AED) treatment [18]. This makes information on the factors associated with increased or decreased seizure likelihood potentially of considerable value for those with ID. Second, while it is quite possible that the situations perceived to be associated with altered likelihood of seizure occurrence for those with ID are the same as those reported for those without, this is not known and the topic remains unexplored. Third, there are a number of well-defined, genetically determined neurodevelopmental syndromes in which both epilepsy and ID are important features. The nature of associated epilepsy often differs between syndromes of ID, and research efforts are increasingly seeking to explain relationships between specific genetic disturbances and the development of seizures in these syndromes [19]. This work raises the question of whether different situations may be associated with increased or decreased likelihood of seizure occurrence in those with epilepsy and ID of different etiologies.

In this study, we sought to describe situations considered to be associated with increased or decreased likelihood of seizure occurrence in individuals with different etiologies of ID. Rett syndrome (RS) and fragile X syndrome (FXS) were the genetic syndromes chosen for investigation. For both, (a) epilepsy forms an important aspect of the syndrome, (b) the syndrome has a well-defined genetic cause, and (c) charities representing the syndrome were able to assist with distributing information about the study. In addition, information was collected a group of people with ID and epilepsy who did not have a diagnosis of one of these syndromes but who had a range of other etiologies of ID or whose etiology was unknown.

Rett syndrome is a rare neurodevelopmental disorder primarily affecting girls. Development over the first 6 months of life often appears normal, but following this, symptoms including social withdrawal, communication difficulty, loss of vocabulary, severe cognitive impairment, stereotypic hand movements, and impaired locomotion emerge [20]. Rett syndrome is caused by mutations in the X-linked gene *MeCP2* [21], whose product is involved in both repression and activation of transcription of many genes [22] and which has been found to regulate formation of glutamatergic synapses early in development [23]. Fragile X syndrome, the most commonly inherited form of intellectual disability, affects males more severely than females and is characterized by mild to profound ID, hyperactivity, behavioral stereotypies, perseverative language, social anxiety, and a number of distinctive physical features [24]. It is caused by mutation of the *FMR1* gene located on the X chromosome, in most cases involving expansion of a trinucleotide (CGG) repeat [25]. The protein coded for by this gene has widespread effects as a translation regulator in neurons, with a particularly important role in the development of neurons and synapses as well as synaptic plasticity [26]. Epilepsy is common in both syndromes; a recent review indicated that estimates of prevalence of seizures range from 60 to 94% in RS and from 12 to 17% in FXS [19].

The primary aim of this study was to identify the situations associated with (a) increased and (b) decreased likelihood of seizure occurrence in those with RS, FXS, and other etiologies of ID and epilepsy. Throughout this paper, situations of increased seizure likelihood and situations of decreased seizure likelihood are abbreviated to SISLs and SDSLs, respectively. The secondary aim was to compare the three etiological groups studied with respect to the nature and frequency of SISLs and SDSLs reported by carers.

Situations of increased seizure likelihood and SDSLs include conditions or circumstances that elsewhere may be described as seizure triggers, seizure precipitants, or seizure inhibiting factors. We chose to use the broader terms SISL and SDSL for three reasons. First, there is disagreement among the research community regarding how terms such as ‘precipitant’ are defined [27]. Second, we wanted to avoid jargon in the questionnaire in order to maintain a clear focus on the phenomenon in question, unaffected by how participants may have interpreted terms such as ‘precipitant’, ‘trigger’, and ‘inhibitor’. Third, we wanted to avoid asking carers to make causal judgements, preferring them to report observed associations which may more closely reflect true events.

## 2 Methods

### 2.1 Overview of method

Three different groups of people with epilepsy and ID were investigated: (i) a group with Rett syndrome (RS), (ii) a group with fragile X syndrome (FXS), and (iii) a group whose ID were associated with a range of other etiologies, excluding RS and FXS. Using a questionnaire, we asked carers of people in these groups to identify specific situations in which they considered that the person they provided care for was either more likely or less likely to have a seizure.

### 2.2 Questionnaire development

The literature was reviewed in order to identify existing surveys on the topic of SISLs and SDSLs. Existing surveys were then reviewed to create a list of situations that may be regarded as possible SISLs or SDSLs. Situations were drawn from those listed in previously used questionnaires as well as those named by respondents in open questions (see ‘Supplementary data 1: Surveys reviewed’ for sources from which the list of situations was drawn). Based on this list, a questionnaire was drafted. Pilot versions were discussed with those involved in the support of people with epilepsy and ID including epilepsy specialist nurses working in ID services, paid and family carers of adults with ID and epilepsy, lay reviewers from the charity Epilepsy Action, and experts on RS, following which, the final version of the carer questionnaire was developed.

The literature was reviewed in order to identify existing surveys on the topic of SISLs and SDSLs. Existing surveys were then reviewed to create a list of situations that may be regarded as possible SISLs or SDSLs. Situations were drawn from those listed in previously used questionnaires as well as those named by respondents in open questions (see ‘Supplementary data 1: Surveys reviewed’ for sources from which the list of situations was drawn). Based on this list, a questionnaire was drafted. Pilot versions were discussed with those involved in the support of people with epilepsy and ID including epilepsy specialist nurses working in ID services, paid and family carers of adults with ID and epilepsy, lay reviewers from the charity Epilepsy Action, and experts on RS, following which, the final version of the carer questionnaire was developed.

### 2.3 Questionnaire structure

The questionnaire can be found in ‘Supplementary data 2: Questionnaire’. It was designed to be completed by family or paid carers who knew the participant well. The first part of the questionnaire concerned clinical and demographic characteristics. In this section, respondents were also asked how many seizure types the person has and asked to describe each seizure type (giving the name of the seizure type if known). If the person had more than four seizure types, the four most common were described.

The questionnaire can be found in ‘Supplementary data 2: Questionnaire’. It was designed to be completed by family or paid carers who knew the participant well. The first part of the questionnaire concerned clinical and demographic characteristics. In this section, respondents were also asked how many seizure types the person has and asked to describe each seizure type (giving the name of the seizure type if known). If the person had more than four seizure types, the four most common were described.

Respondents then answered questions about each of the person's seizure types in turn. A list of 57 situations was provided, and the respondent was asked to tick, for each seizure type, whether each item on the list was associated with (a) decreased chance of a seizure (indicating an SDSL), (b) no association with seizure occurrence, or (c) increased chance of a seizure (indicating an SISL). Respondents were also asked to name, as a free text response, any additional SISLs and SDSLs.

Epileptic seizures can be particularly difficult to distinguish from other paroxysmal events or behaviors in those with ID [28–30]. For each seizure type, the carer was asked to indicate whether the treating clinician had said (a) the seizure type is epileptic, (b) she/he is uncertain whether or not it is epileptic, (c) it is not epileptic, or (d) none of these. To maximize the likelihood that all data analyzed related to epileptic events, we excluded from the analysis all data concerning seizure types where any option other than (a) was endorsed.

### 2.4 Participants and ethical approval

Eligible carers were 18 + and currently supporting a child or adult with epilepsy and ID and at least 1 seizure in the last year. Carers had to have known the person for at least one year and be the carer who knew the person best.

The questionnaire was distributed via charities representing people with epilepsy and people with the genetic syndromes of interest. The survey was distributed in postal form (via Rett UK and The Fragile X Society (UK), to families of people with these conditions). In addition, an online version was advertised on charity websites, social media, and newsletters (via National Fragile X Foundation (USA), Epilepsy Foundation (Australia), Epilepsy Action UK, and the UK Health and Learning Disability Network). Subsequent reminders were included in the newsletters of the charities concerned where possible in an effort to maximize the number of responses. Although it was clear to potential participants that the study was about the situations in which seizures occur, the recruitment material encouraged all eligible carers to participate, whether or not they believed there to be any situations associated with increased or decreased seizure occurrence. Where the survey was distributed to members of the syndrome specific charities, carers were not asked to report on the etiology of the ID which was assumed to be the specific genetic pathology. The study was approved by the University of Cambridge Psychology Research Ethics committee, and all participants gave written informed consent.

### 2.5 Analysis: coding of seizure descriptions

Carer descriptions of seizures believed to be epileptic in nature, on the basis of the treating clinician's opinion (as detailed in Section 2.3), were categorized by a consultant neuropsychiatrist with experience of managing epilepsy in people with ID (HR), blind to etiology and other participant characteristics. Based on carer descriptions, seizures were categorized as either of focal onset or generalized onset, and where the information given was insufficient for such characterization, seizures were coded as of unknown onset. For respondents for whom all seizure types described could be categorized, the person with ID was then classified according to whether the carer's seizure descriptions suggested focal-onset seizures only, generalized-onset seizures only, or both focal-onset and generalized-onset seizures.

### 2.6 Analysis: statistical tests

The main outcome variables were the number and nature of SISLs and SDSLs reported by the carer, summarized across all the person's eligible seizure types (up to a maximum of four), including responses from the questionnaire tick list and free text responses. If a respondent endorsed a particular SISL or SDSL for more than one of the person's seizure types, this was only counted once. Four types of analysis were included in this work and are detailed as follows.

Statistical comparisons were made between the three etiological groups with respect to participant characteristics. For univariate between-group comparisons of nominal outcome variables, chi-square and Fisher's exact (when expected cell counts were < 5) tests using the CROSSTABS procedure in SPSS [31] were used. Kruskal–Wallis (K–W) tests were used for between-group comparisons of ordinal and skewed interval outcome variables.

Log-linear regression [32] using the GENLIN procedure in SPSS [31] was used to investigate associations between multiple characteristics of the respondent or the person with ID (for example, the person's seizure frequency) and number of SISLs reported by carers.

Chi-square tests were used to investigate whether carers of people with different etiologies of ID differed with regard to how commonly particular SISLs of interest were reported.

Finally, multidimensional scaling (MDS) [33] was used to assess relationships between reported SISLs in order to identify possible clusters of SISLs, that is, groups of SISLs that tend to co-occur in carers' responses. Each carer's response to each situation listed on the questionnaire was categorized as either 1 (the carer considered the situation an SISL) or 0 (the carer considered there to be no association or considered it an SDSL), and the analysis was subsequently carried out using the PROXSCAL procedure in SPSS [31].

Alpha was set at 0.05 with Bonferroni correction for multiple comparisons applied where appropriate.

## 3 Results

The results are presented as follows. First, characteristics of respondents and the people with ID they supported are provided. Following this, the mean numbers of SISLs and SDSLs reported by carers in each of the three groups are provided, and the participant characteristics which predict the number of SISLs reported are identified. The nature of SISLs and SDSLs reported by carers in each of the three groups are presented and compared, and the data are analyzed for clustering of commonly reported SISLs.

### 3.1 Participant characteristics

Participant characteristics for the 100 eligible responses received are reported in Table 1. Ninety percent of these responses were from the UK charities. The groups differed significantly with regard to (i) gender of the person with ID, *Χ^2^*(2) = 61.8, p < 0.001; (ii) number of seizure types, Kruskal–Wallis (K–W) *Χ^2^*(2) = 14.3, p = 0.001; (iii) seizure frequency, K–W *Χ^2^*(2) = 12.1, p = 0.002; (iv) ID severity, K–W *Χ^2^*(2) = 44.1, p < 0.001; (v) types of seizure the person has, Fisher's exact, p = 0.004; and (vi) age at seizure onset, K–W *X^2^*(2) = 9.1, p = 0.010.

### 3.2 Number of SISLs and SDSLs reported

**Fig. 1:**
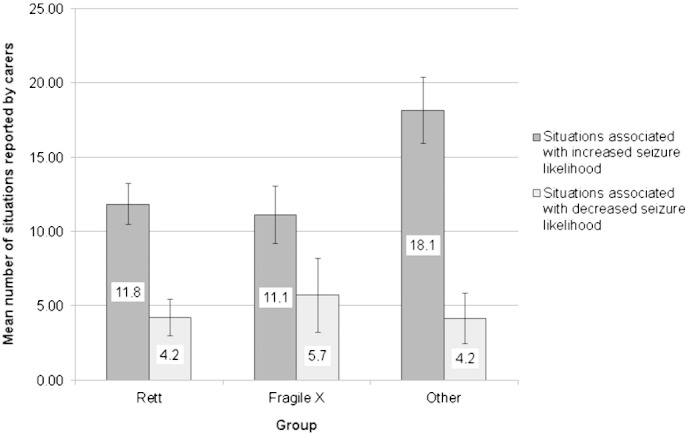
Number of situations reported to be associated with increased or decreased seizure occurrence by etiological group. Error bars indicate standard error.

Ninety-eight percent of the respondents reported at least one SISL, and 60% reported at least one SDSL, with two percent reporting neither. Fig. 1 shows the mean number of different SISLs and SDSLs reported by respondents in each group, including both those identified from the tick list in the questionnaire and those named in response to the open questions that followed the tick list. However, most of these were from the tick list, with very few (2.4% of all SDSLs reported and 3.0% of all SISLs reported) arising from responses to the open questions.

The mean numbers of SISLs and SDSLs reported by carers in each of the three groups were compared statistically. With regard to SDSLs, there was no significant difference in number reported between the three etiological groups, K–W *Χ^2^*(2) = 0.23, p = 0.890. However, with regard to SISLs, there was a significant difference between groups, K–W *Χ^2^* (2) = 8.62, p = 0.013, with fewer SISLs reported in the RS and FXS groups than in the ‘other etiologies’ group. However, because etiological groups were found to differ with regard to various characteristics of the person with ID (see Section 3.1), log-linear regression (including a scale factor for overdispersion [34]) was conducted, with number of SISLs reported as the dependent variable and etiological group, gender of the person with ID, seizure frequency, number of seizure types, age at first seizure, and ID severity as independent variables. The seizure types the person has (focal-onset seizures only, generalized-onset seizures only, or both focal-onset and generalized-onset seizures), as suggested by the carer's seizure descriptions, was not included in the initial model because this information was unavailable for 28% of the sample, thus reducing the sample size for the regression. More seizure types (Wald *X^2^*(1) = 8.20, p = 0.004) and a greater seizure frequency (Wald *X^2^*(3) = 8.64, p = 0.035) were both associated with a greater number of SISLs reported by the carer. Etiological group (Wald *X^2^*(2) = 2.73, p = 0.26), ID severity (Wald *X^2^*(2) = 1.64, p = 0.44), age at first seizure (Wald *X^2^*(1) = 0.14, p = 0.71), and gender of the person with ID (Wald *X^2^*(1) = 0.08, p = 0.77) were not significant predictors of the number of SISLs reported. Evaluating the model as a whole, we found that the Bayesian Information Criterion (BIC) for the model including the variables listed was 932.89, while the BIC for the intercept-only model was 1084.46. Using the cutoffs reported by Jones and colleagues [35] these BIC values indicate the model is a good fit compared with the intercept-only model.

The regression was run for a second time including only seizure frequency and number of seizure types with the addition of the variable indicating whether the carer's seizure descriptions suggested focal-onset seizures only, generalized-onset seizures only, or both focal-onset and generalized-onset seizures. The former two remained significant predictors in this smaller model, but the type of seizure was not a significant predictor (Wald *Χ^2^*(2) = 1.91, p = 0.39). Again, the model was a substantially improved fit, with a BIC of 720.24 compared with 860.95 for the intercept-only model.

In considering the association with number of seizure types, it is important to remember that in the questionnaire, carers were asked to report SISLs for each seizure type in turn. Thus, those supporting someone with multiple seizure types might be expected to report a greater number of SISLs simply by the design of the questionnaire, as they had viewed the list of situations multiple times and had multiple opportunities to endorse these situations. To explore this, for the up to four seizure types that were investigated in the questionnaire, the number of novel SISLs from the tick list that were endorsed that had not been reported by the carer for any of the previously investigated seizure types was ascertained. The mean number of novel SISLs reported for each of seizure types 1–4 was calculated. In calculating the means, only relevant respondents were included (so, for example, in calculating this for seizure type 4, only people with a fourth seizure type were included). The mean number of SISLs that carers in each of the three groups reported for seizure type 1 lay between 8.6 and 14.5 SISLs, while for seizure types 2–4, the mean number of novel SISLs reported lay between 0 and 2.8 SISLs. This indicates that of the SISLs that carers reported, very few were reported for subsequent seizure types that had not already been reported for the first seizure type investigated.

### 3.3 Nature of SISLs and SDSLs reported

For each etiological group, the percentage of carers reporting each SISL was calculated, including situations from the tick list and open question. Overall, the most commonly reported SISL was illness, which was reported by 71% of the whole sample.

The ten most commonly reported SISLs for each group are listed in Table 2 (where necessitated by multiple SISLs having equal prevalence, more than ten are listed). Percentages for menstruation include females only.

The percentage of carers reporting each SISL were compared statistically between the groups for SISLs that were among the top five most commonly reported for at least one of the groups (illness, tiredness or drowsiness, during the night, waking up, menstruation, during the evening, relaxation, stress, sleep deprivation, and during sleep). Chi-square tests for each (excluding menstruation where group differences may be attributed to gender difference between groups) were conducted with Bonferroni correction. Significant associations between group and whether the SISL was endorsed were found for (i) tiredness/drowsiness, *Χ^2^*(2) = 10.6, p = 0.005; (ii) sleep deprivation, *Χ^2^*(2) = 21.1, p < 0.001; and (iii) during sleep, *Χ^2^*(2) = 11.3, p = 0.004. Other SISLs analyzed did not show a significant association with group, with all p-values greater than the Bonferroni-corrected alpha of 0.0056. Examination of the standardized residuals from the chi-square tests [36] indicated that for all three significant results, the association between etiological group and whether the situation was endorsed was driven by participants in the ‘other etiologies’ group reporting the SISL in question significantly more than expected. In agreement with this, Bonferroni-corrected post hoc chi-square tests comparing the RS and FXS groups only did not show any significant differences between groups (tiredness/drowsiness *Χ^2^*(1) = 0.71, p = 0.40; sleep deprivation *Χ^2^*(1) = 0.75, p = 0.39; during sleep *Χ^2^*(1) = 0.26, p = 0.87).

Multidimensional scaling was conducted using data combined from all three etiological groups on the SISLs that were in the top ten most commonly reported for at least one of the three groups. Stress 1 (the recommended measure of goodness of fit [37]) was 0.31, representing poor fit [38]. Therefore, there was no evidence of clustering of reported SISLs.

There was little consensus within groups with regard to SDSLs, with no situation being reported as associated with decreased likelihood of seizure occurrence by a large proportion of any group. Table 3 shows the most commonly reported for each group, limited to those reported by at least 20% of carers in the group. These results are reported for completeness, but because of the small sample sizes involved, they should be interpreted with caution.

## 4 Discussion

To our knowledge, this is the first published survey of environmental, emotional, or physiological situations associated with increased (SISLs) or with decreased (SDSLs) seizure likelihood in people with ID and treatment-refractory epilepsy. We have reported the situations associated with seizure occurrence, as identified by carers, for those with epilepsy and ID of various etiologies. The potential importance of this work is that identification of SISLs or SDSLs could inform nonpharmacological approaches to seizure management that may provide a useful adjunct to antiepileptic drug treatment. However, it is important to note that the study concerns carer-reported situations only, which may or may not reflect true associations with increased or decreased seizure likelihood. The purpose of the study was to identify carer-perceived SISLs and SDSLs, providing a starting point for future research on this topic in those with ID and epilepsy.

### 4.1 Nature of SISLs reported

Tiredness, stress, and sleep deprivation (the most commonly reported SISLs in the general population of those with epilepsy) did feature highly as SISLs in this investigation, as did sleep and transitions between sleep and wakefulness. This indicates some important similarities between those with ID and those without ID. Interestingly however, stress was not the most commonly reported SISL in the current investigation. Stress can be difficult to define and identify in people with ID depending on their communication skills, and the meaning or experience of ‘stress’ is likely to vary depending on the severity of the person's ID, which might explain this difference.

Instead, in contrast to previous research in those without ID, illness was the most common SISL in our sample. Considering previous reports in the literature relating to those with ID, in one survey including people with ID, illness was the most commonly reported precipitant at 32% [39] and the second most common at 27% in a survey of a pure ID adult sample [40], although another survey including those with ID found it to be of low prevalence [11]. The high prevalence found in the current investigation suggests that this is an important SISL for this group. In the management of treatment-refractory seizures, clinicians should be alert to symptoms of possible concomitant physical illnesses which might be impacting on seizure control. This may be particularly important for those with ID, given that a number of health problems are relatively common in this group [41]. We did not ask carers to specify the nature of the illnesses which they considered to be associated with increased seizure likelihood. The particular illness states associated with increased seizure likelihood, as well as possible mechanisms of these associations, would be important topics for future research.

### 4.2 Number of carers reporting at least one SISL

In the current study, almost all participants (98%) reported at least one SISL. The literature suggests that 53–97% of people with epilepsy [1–5,8,10] and 80–89% of carers or other informants [3,12] report at least one seizure precipitant. With regard to those with ID, there is little research available. However, Cull and colleagues [11] conducted a survey of young people in special residential schools, many of whom had ID. Students and also staff members who knew the student well were surveyed, and it was found that 63.3% of the students and 56.9% of the staff members reported at least one precipitant. The staff members were more likely to report precipitants for those students with IQs in the average range (those without ID). In another investigation that included people with ID, 62% of children with epilepsy were reported to have at least one precipitant, and those with precipitants were more likely to have neurological deficits, with 84% of those with severe ID having precipitants reported [39]. These investigations present conflicting findings on the effect of ID on precipitant prevalence, and further research comparing those with ID and those without ID within a single investigation may be informative. The high prevalence in the current study may be the result of a selection bias in that those carers with an SISL to report may have been more likely to complete the questionnaire. It may also be a result of the terminology used. Carers were asked to report when seizures are more likely to occur without requiring them to make a causal judgment about the observed association. Carers might report certain situations as SISLs which they would not report as precipitants or triggers as these latter terms imply a causal element about which the carer may be unsure.

### 4.3 Number of SISLs reported per carer

The mean number of SISLs reported was high for all groups and considerably higher than the mean numbers of precipitants reported in previous research, which range from 1.22 to 6.57 [5,8,12]. Again, this may be a consequence of differences in survey methodology and terminology. Greater seizure frequency and more seizure types were associated with a greater number of SISLs being reported. Evidence from previous research regarding this relationship is mixed; some surveys found an association between reporting of SISLs and poor seizure control [3,7,8], while others did not [6,10,39]. Both the number of seizure types and seizure frequency may be considered markers of epilepsy severity. A speculative interpretation of the association between these variables and the number of SISLs is that it could be a result of those with more severe epilepsy being constantly close to the “seizure threshold”, so that only a mildly provoking situation would be sufficient to precipitate a seizure. An alternative explanation might be that it is not a feature of the epilepsy severity per se but how this severity impacts upon the carer and his or her judgements regarding seizure occurrence. For those with high seizure rates, carers have more opportunities to observe the situations of seizure occurrence and, therefore, may be more able to identify associations than if seizure events are rare, or it might be the case that in those with more severe epilepsy, carers are more affected by a feeling of lack of control and a need to understand seizure events and are, thus, more prone to seek explanations for seizures that may or may not reflect true relationships between certain situations and seizure occurrence. This explanation receives some support from the finding that high anxiety levels, as well as low levels of belief in the power of others (e.g., physicians) to influence health status, are associated with increased reporting of precipitants by patients with epilepsy [8].

### 4.4 Comparisons between etiological groups

Interestingly, there was considerable similarity between the three etiological groups studied with regard to SISLs. When taking into account other participant characteristics, there were no statistically significant between-group differences in the number of SISLs reported. Furthermore, the most commonly reported SISLs in each group were fairly similar. The only statistically significant between-group differences in SISLs reflected differences between the ‘other etiologies’ group and the two genetic syndrome groups rather than differences between the FXS group and the RS group. This is despite substantial differences in the pathophysiology of these syndromes. This suggests that SISLs might act on a component of the seizure generation process downstream from the primary etiology, relating to a process of seizure initiation that is common across different etiologies. Alternatively, remembering that this investigation focuses on carer report, the similarities may reflect shared beliefs about seizure occurrence not necessarily reflective of actual patterns of association. This seems unlikely, however, given that carers of those with the two syndromes will likely have quite different life experiences. Interestingly, the statistically significant differences that were found all related to sleep (sleep deprivation, tiredness/drowsiness, and during sleep were all reported as SISLs more commonly in the ‘other etiologies’ group than the two syndrome groups). Given the range and prevalence of sleep problems in those with RS [42–46], FXS [47–49], and ID in general [50–53], further research focusing on the sleep–wake cycle and its relationship to seizures in those with ID and epilepsy may be warranted.

### 4.5 SDSLs

Situations of decreased seizure likelihood were less commonly reported than SISLs in agreement with previous research [1,6]. This might be explained by the circumstances surrounding seizure events being more salient than those surrounding a lack of such events. Because no SDSL was reported by a large proportion of the sample, firm conclusions about the situations when seizures are less likely to occur cannot be drawn from the current data.

### 4.6 Representativeness of the sample

Finally, it is important to consider the extent to which the results from our sample are representative of the epilepsy experienced by the wider populations of those with FXS and RS. Comparison of the characteristics of the epilepsy of our sample (seizure frequency and age at onset) with existing data on the epilepsy of those with FXS and RS provides some indication of this.

The seizure frequencies reported in our samples were higher than those reported in previous studies. The percentages of patients with RS and epilepsy with daily and weekly seizures were 11% and 20%, respectively, in a recent study of 602 people with RS [54] compared with 34% and 25%, respectively, in our sample. In a large study of 1394 people with FXS; 69% of males with seizures and 89% of females with seizures had not had a seizure in the last 6 months [55], again indicating a lower seizure frequency than in our sample. Unlike these studies, we excluded those who had not had any seizures in the last year, which will have made a significant contribution to these differences.

Age at first seizure was also considered, and our results were found to be similar to those of previous studies. The mean age at first seizure was 4.5 years in our group with RS and 6.2 years in our group with FXS. The mean age at onset of seizures in those with RS was found to be 4.7 years in a multicenter study of 165 people with RS [56], while in those with FXS, the most common age at seizure onset has been reported to be between 4 and 10 years [55], consistent with the data from our sample.

### 4.7 Limitations

The study has several limitations. First, response rates are unknown, and while we cannot be certain of the extent to which our samples were representative of the populations under investigation, there is evidence that those with high seizure frequencies were overrepresented in this study, although age at onset of seizures in our sample was similar to that reported in other studies. Second, the sample size of 100, while substantial given the dispersal of eligible participants and comparable with other surveys [1,3,6,10–12], is relatively small, and for clarification of these findings, larger scale studies are needed. Third, though steps were taken to ensure that the data collected and analyzed referred to epileptic seizures only, it is possible that SISLs and SDSLs for some nonepileptic events may have been identified. Fourth, etiologies of ID in the ‘other etiologies’ group were unknown for many of the individuals concerned, making it difficult to characterize this group. Fifth, the use of a tick list on the questionnaire (rather than just an open question) may have resulted in carers reporting SISLs and SDSLs that they would not have reported had they not been prompted in this way, potentially inflating the number of situations reported. Sixth, while there is evidence that reported precipitants differ depending on seizure type and epilepsy syndrome [4,5,10], this was not investigated in the current study. This was not attempted because information available on seizure types was in the form of witness descriptions only, which may be inaccurate [57,58]. Finally, it is unknown to what extent carers' reports of SISLs and SDSLs reflect true objectively observable relationships between these situations and seizure occurrence. This is an ongoing limitation in this area, with the majority of evidence coming from self-report and carer-report surveys. Prospective studies are needed, in which occurrences of seizures and of possible trigger events are reported contemporaneously in carer diaries, allowing a more objective assessment of possible relationships between certain situations and increased likelihood of seizure occurrence. In accordance with this need, a prospective investigation in people with epilepsy and ID is currently being undertaken by the authors.

## 5 Conclusions

As well as potentially offering insights into the mechanisms underlying seizure generation, the study of SISLs and SDSLs is of considerable clinical relevance as a greater understanding of the circumstances in which even just a minority of an individual's seizures occur may ultimately lead to improved epilepsy management using behavioral strategies of seeking or avoiding certain situations depending on when seizures are most likely to occur. This may be particularly valuable for those with ID, given the considerable proportion of people who continue to have seizures despite AED treatment.

Supplementary data 1 Surveys reviewed. Literature that was reviewed in developing the questionnaire used in this study.

Supplementary data 2 Questionnaire. The questionnaire used for data collection.

Supplementary data to this article can be found online at http://dx.doi.org/10.1016/j.yebeh.2014.08.016.

## Figures and Tables

**Table 1:** Participant characteristics. Data are percentages of sample or mean values with standard deviation in parentheses.

	Rett syndrome N = 44	Fragile X syndrome N = 25	Other etiologies N = 31
Age of carer	49.6 (9.4)	50.7 (12.1)	46.1 (11.6)
Years they have known person with ID	19.5 (9.7)	19.1 (11.0)	16.1 (12.3)
Gender of carer	11.4% males	16.0% males	9.7% males
Relationship to person with ID			
Relative	98.0%	100.0%	94.0%
Paid carer	2.0%	0.0%	6.0%
Age of person with ID (years)	19.3 (9.2)	19.2 (11.0)	19.0 (16.1)
Gender of person with ID	4.5% males	100.0% males	58.1% males
Age at first seizure (years)	4.5 (0.6)	6.2 (1.1)	3.7 (1.0)
Number of seizure types	2.0 (0.9)	2.3 (1.6)	3.1 (1.4)
Number of AEDs	1.6 (0.8)	1.7 (0.9)	2.0 (1.1)
Reported seizure types			
Data unavailable	20.5%	56.0%	16.1%
Focal-onset seizures only	20.5%	28.0%	6.5%
Generalized-onset seizures only	40.9%	8.0%	35.5%
Both focal-onset and generalized-onset seizures	18.2%	8.0%	41.9%
ID severity			
Mild	0.0%	0.0%	29.0%
Moderate	0.0%	52.0%	42.0%
Severe or profound	100.0%	48.0%	29.0%
Seizure frequency			
Less than 1 per month	15.9%	32.0%	16.1%
Monthly^a^	25.0%	48.0%	12.9%
Weekly^b^	25.0%	12.0%	35.5%
At least 1 per day	34.1%	8.0%	35.5%

AED = antiepileptic drug.

a At least 1 per month but less than 1 per week.

b At least 1 per week but less than 1 per day.

**Table 2:** Most commonly reported SISLs for each etiological group. Listed in descending order of prevalence.

Rett syndrome N = 44		Fragile X syndrome N = 25		Other etiologies N = 31	
SISL	% of respondents	SISL	% of respondents	SISL	% of respondents
1. Illness	81.8	1. Illness	52.0	1. Tiredness/drowsiness	83.9
2. Tiredness/drowsiness	54.5	2 = During the evening	44.0	2. Sleep deprivation	74.2
3 = During the night	47.7	2 = During the night	44.0	3 = During sleep	71.0
3 = Waking up	47.7	2 = Relaxation	44.0	3 = Illness	71.0
4. Menstruation	47.6	2 = Stress	44.0	4 = During the night	67.7
5. Constipation	40.9	2 = Tiredness/drowsiness	44.0	4 = Waking up	67.7
6. Stress	38.6	3. During the morning	40.0	5 = When falling asleep	61.3
7. Pain	36.4	4 = Anxiety	36.0	5 = Stress	61.3
8 = When falling asleep	34.1	4 = At home	36.0	6 = At home	51.6
8 = During sleep	34.1	4 = Doing nothing	36.0	6 = Excitement	51.6
		4 = During sleep	36.0		
		4 = Excitement	36.0		
		4 = Waking up	36.0		

**Table 3:** Most commonly reported SDSLs for each etiological group. Listed in descending order of prevalence. Only those with prevalence of at least 20% are shown.

Rett syndrome N = 44		Fragile X syndrome N = 25		Other etiologies N = 31	
SDSL	% of respondents	SDSL	% of respondents	SDSL	% of respondents
1. During the night	29.5	1 = Waking up	20.0	1. Relaxation	22.6
2. Relaxation	27.3	1 = Outdoors	20.0	2. Outdoors	22.6
3. Outdoors	22.7	1 = During the morning	20.0		
		1 = When falling asleep	20.0		
		1 = During sleep	20.0		
