# Supplementary material for: Why do seizures occur when they do? Situations perceived to be associated with increased or decreased seizure likelihood in people with epilepsy and intellectual disability
Source: Epilepsy Behav. 2014 Oct;39:78–84. doi: 10.1016/j.yebeh.2014.08.016 (PMC4274323; doi:10.1016/j.yebeh.2014.08.016)
Supplement: Supplementary data 1 — Surveys reviewed. Literature that was reviewed in developing the questionnaire used in this study. [file mmc1.docx]

Literature reviewed in development of questionnaire

[[1-14](#_ENREF_1)]

[1] Pinikahana J, Dono J. The lived experience of initial symptoms of and factors triggering epileptic seizures. Epilepsy Behav 2009;15: 513-20.

[2] Nakken KO, Solaas MH, Kjeldsen MJ, Friis ML, Pellock JM, Corey LA. Which seizure-precipitating factors do patients with epilepsy most frequently report? Epilepsy Behav 2005;6: 85-9.

[3] Fang PC, Chen YJ, Lee IC. Seizure precipitants in children with intractable epilepsy. Brain Dev 2008;30: 527-32.

[4] Antebi D, Bird J. The facilitation and evocation of seizures. A questionnaire study of awareness and control. Br J Psychiatry 1993;162: 759-64.

[5] Frucht MM, Quigg M, Schwaner C, Fountain NB. Distribution of seizure precipitants among epilepsy syndromes. Epilepsia 2000;41: 1534-9.

[6] Spector S, Cull C, Goldstein LH. Seizure precipitants and perceived self-control of seizures in adults with poorly-controlled epilepsy. Epilepsy Res 2000;38: 207-16.

[7] Sperling MR, Schilling CA, Glosser D, Tracy JI, Asadi-Pooya AA. Self-perception of seizure precipitants and their relation to anxiety level, depression, and health locus of control in epilepsy. Seizure 2008;17: 302-7.

[8] da Silva Sousa P, Lin K, Garzon E, Sakamoto AC, Yacubian EM. Self-perception of factors that precipitate or inhibit seizures in juvenile myoclonic epilepsy. Seizure 2005;14: 340-6.

[9] Spatt J, Langbauer G, Mamoli B. Subjective perception of seizure precipitants: results of a questionnaire study. Seizure 1998;7: 391-5.

[10] Cull CA, Fowler M, Brown SW. Perceived self-control of seizures in young people with epilepsy. Seizure 1996;5: 131-8.

[11] Fisher RS, Vickrey BG, Gibson P, Hermann B, Penovich P, Scherer A, Walker S. The impact of epilepsy from the patient's perspective I. Descriptions and subjective perceptions. Epilepsy Res 2000;41: 39-51.

[12] Hayden M, Penna C, Buchanan N. Epilepsy: patient perceptions of their condition. Seizure 1992;1: 191-7.

[13] Mayville EA. The Matson and Mayville (M&M) seizure scale: An assessment of psychological and environmental variables contributing to seizure activity in persons with mental retardation. In: Unpublished doctoral dissertation, Louisiana State University; 2001.

[14] Verduyn CM, Stores G, Missen A. A survey of mothers' impressions of seizure precipitants in children with epilepsy. Epilepsia 1988;29: 251-5.
