# Supplementary material for: Why do seizures occur when they do? Situations perceived to be associated with increased or decreased seizure likelihood in people with epilepsy and intellectual disability
Source: Epilepsy Behav. 2014 Oct;39:78–84. doi: 10.1016/j.yebeh.2014.08.016 (PMC4274323; doi:10.1016/j.yebeh.2014.08.016)
Supplement: Supplementary data 2 — Questionnaire. The questionnaire used for data collection. [file mmc2.pdf]

UNIVERSITY OF CAMBRIDGE

epilepsy action

Funded by an Epilepsy Action research grant

The **CARES** Questionnaire:

Circumstances **A**ffecting the **R**isk of having an **E**pileptic **S**eizure

This questionnaire is for people who care for or support somebody who has epilepsy and a learning disability. Please fill in the questionnaire according to what you know about the person you care for or support and his/her epilepsy.

Office use only: *Participant ID number……………………*

Part A: About you and the person you support

Q1. What is your relationship to the person with epilepsy and learning disability? Please tick:
Family member ☐ Paid support worker/carer ☐ Other ☐-If so please state……………………

Q2. What is your gender? Please circle: M / F

Q3. What is your age? ....years

Q4. How many years have you known the person with epilepsy and learning disability? ………………years

Q5. What is the gender of the person with epilepsy and learning disability? Please circle: M / F

Q6. What is his/her age? ....years ....months

Q7. In addition to epilepsy, has s/he also been diagnosed with non-epileptic seizures? Please circle: Y / N

Q8. Please list below all medications s/he is currently taking:

……

……

………..

Q9. (a) Do you feel that s/he is able to reliably describe his/her physical symptoms and feelings to you?
Please tick: Yes ☐ No ☐ Don't know ☐

(b) If No or Don't Know, is s/he able to show you in other, non-verbal ways?
Please tick: Yes ☐ No ☐ Don't know ☐

Q10. How severe is his/her learning disability? Please tick:
Mild ☐ Moderate ☐ Severe/profound ☐ Don't know ☐

Q11. If you know why s/he has a learning disability, please could you provide as much detail as you can, including any relevant diagnoses:

……

……

………..

Q12. How old was s/he when s/he had the first seizure? ……….years ………months

Q13. For some people, all their epileptic seizures are the same, while for others there may be a number of different seizure types. How many different epileptic seizure types does the person you care for have? Please write the number of seizure types here:……………

Q14. For each seizure type s/he has, please **describe it as fully as you can**. Think back to what you have **observed** during these seizures and try to describe **everything that happens** during the seizure. (Also, if you know the name of this type of seizure, please add that to your description.) If s/he has more than 4 seizure types, please choose the 4 that occur most often. Please describe the most frequent type as seizure type 1, followed by the second most frequent type, and so on.

Seizure type 1: ……

……

……

……

……

Seizure type 2: ……

……

……

……

……

Seizure type 3: ……

……

……

……

……

Seizure type 4: ……

……

……

……

……

Q15**. Including all the different seizure types s/he has**, how often does s/he have a seizure? Answer this by thinking back over the last year. Please tick one:

- Less often than once a month☐– if so how many seizures over the past year?.........
- About once a month☐
- More often than once a month, but less often than once a week☐
- About once a week☐
- More often than once a week, but less often than once a day☐
- About once a day☐
- More often than once a day☐- if so how many per day?............

In Parts B, C, D and E, you will be asked separately about each of the seizure types you listed in Q14. How many of these Parts you complete will depend on how many seizure types you listed in Q14. The instructions at the bottom of each page will guide you where to go next so you only answer Parts relevant to you. Please now go to Part B.

Part B: Seizure type 1

For the seizure type you described as **'Seizure type 1'** in Question 14, please answer the following questions.

Q16. Some situations are listed below. Please go through **both** lists and tick one column for each situation, to say **whether the situation is associated with** a **decreased** or **increased chance** of the seizures you described as '**Seizure type 1**', in the person you care for. If the situation is not related to whether seizures are likely to occur, please tick 'no association'. If a situation is not applicable to the person you care for, leave it blank and move on to the next one.

**Situation**	Seizures **less likely**	No association	Seizures **more likely**
Example		√	
Outdoors			
Excitement			
Relaxed			
In noisy environment			
Ill (e.g. with fever, bug)			
During the night			
At work			
Stress			
Alone			
Thirsty or hungry			
Tired or drowsy			
Walking or other exercise			
Frustrated			
In crowded room			
Sudden change in posture			
When waking up after sleep			
Alert			
With a stranger			
During the afternoon			
Surprised/startled			
Being criticised/reprimanded			
Engaged in work or education activity			
Sudden change in light levels			
Waiting for an activity/task to begin			
Visiting relative or friend's house			
Frightened			
When falling asleep			
Washing/dressing			

**Situation**	Seizures **less likely**	No association	Seizures **more likely**
Angry			
Menstruating			
Winding down after stress			
Engaged in leisure activity			
Constipated			
Touch/tactile stimulation			
During the evening			
At home (main residence)			
Too cold or too hot			
Flickering light			
At day activities/day centre			
Toward the end of an activity/task			
Hyperventilating/breathing abnormally			
TV or computer games			
Away on holiday			
Sudden noise			
Pain			
Just after an activity/task has finished			
Bored			
During the morning			
Sitting still			
Doing nothing			
Sad			
With friends or family			
Sleep deprived			
Happy			
During sleep			
Anxious			
Reading			

Q17. (a) Is there anything **not included in the two lists above** that **increases** the likelihood of seizures of this type in the person you care for? If so please write this here:……………………………………………………………………………

(b) Is there anything **not included in the two lists above** that **decreases** the likelihood of seizures of this type in the person you care for? If so please write this here:……………………………………………………………………………

Q18. What has the doctor treating his/her epilepsy said about this seizure type? Please tick one:

The doctor has said this seizure type is epileptic ☐
The doctor has said s/he is uncertain about whether this seizure type is epileptic ☐
The doctor has said this seizure type is not epileptic ☐
None of the above ☐

Q19. How often does s/he have a seizure **of this type**? Answer this by thinking back over the last year. Please tick 1:

Less often than once a month ☐– if so how many seizures over the past year?..........
About once a month ☐
More often than once a month, but less often than once a week ☐
About once a week ☐
More often than once a week, but less often than once a day ☐
About once a day ☐
More often than once a day ☐ - if so how many per day?............

--- If you listed more than one seizure type in question 14, please continue to part C. If not, go to part F ---

Part C: Seizure type 2

For the seizure type you described as **'Seizure type 2'** in Question 14, please answer the following questions.

Q20. Some situations are listed below. Please go through **both** lists and tick one column for each situation, to say **whether the situation is associated with** a **decreased** or **increased chance** of the seizures you described as '**Seizure type 2**', in the person you care for. If the situation is not related to whether seizures are likely to occur, please tick 'no association'. If a situation is not applicable to the person you care for, leave it blank and move on to the next one.

**Situation**	Seizures **less likely**	No association	Seizures **more likely**
Example		√	
Outdoors			
Excitement			
Relaxed			
In noisy environment			
Ill (e.g. with fever, bug)			
During the night			
At work			
Stress			
Alone			
Thirsty or hungry			
Tired or drowsy			
Walking or other exercise			
Frustrated			
In crowded room			
Sudden change in posture			
When waking up after sleep			
Alert			
With a stranger			
During the afternoon			
Surprised/startled			
Being criticised/reprimanded			
Engaged in work or education activity			
Sudden change in light levels			
Waiting for an activity/task to begin			
Visiting relative or friend's house			
Frightened			
When falling asleep			
Washing/dressing			

**Situation**	Seizures **less likely**	No association	Seizures **more likely**
Angry			
Menstruating			
Winding down after stress			
Engaged in leisure activity			
Constipated			
Touch/tactile stimulation			
During the evening			
At home (main residence)			
Too cold or too hot			
Flickering light			
At day activities/day centre			
Toward the end of an activity/task			
Hyperventilating/breathing abnormally			
TV or computer games			
Away on holiday			
Sudden noise			
Pain			
Just after an activity/task has finished			
Bored			
During the morning			
Sitting still			
Doing nothing			
Sad			
With friends or family			
Sleep deprived			
Happy			
During sleep			
Anxious			
Reading			

Q21. (a) Is there anything **not included in the two lists above** that **increases** the likelihood of seizures of this type in the person you care for? If so please write this here:...

(b) Is there anything **not included in the two lists above** that **decreases** the likelihood of seizures of this type in the person you care for? If so please write this here:...

Q22. What has the doctor treating his/her epilepsy said about this seizure type? Please tick one:

The doctor has said this seizure type is epileptic ☐

The doctor has said s/he is uncertain about whether this seizure type is epileptic ☐

The doctor has said this seizure type is not epileptic ☐

None of the above ☐

Q23. How often does s/he have a seizure **of this type**? Answer this by thinking back over the last year. Please tick 1:

Less often than once a month ☐– if so how many seizures over the past year?..........

About once a month ☐

More often than once a month, but less often than once a week ☐

About once a week ☐

More often than once a week, but less often than once a day ☐

About once a day ☐

More often than once a day ☐ - if so how many per day?............

--- If you listed more than two seizure types in question 14, please continue to part D. If not, go to part F ---

Part D: Seizure type 3

For the seizure type you described as **'Seizure type 3'** in Question 14, please answer the following questions.

Q24. Some situations are listed below. Please go through **both** lists and tick one column for each situation, to say **whether the situation is associated with** a **decreased** or **increased chance** of the seizures you described as '**Seizure type 3**', in the person you care for. If the situation is not related to whether seizures are likely to occur, please tick 'no association'. If a situation is not applicable to the person you care for, leave it blank and move on to the next one.

**Situation**	Seizures **less likely**	No association	Seizures **more likely**	**Situation**	Seizures **less likely**	No association	Seizures **more likely**
Example		√		Angry			
Outdoors				Menstruating			
Excitement				Winding down after stress			
Relaxed				Engaged in leisure activity			
In noisy environment				Constipated			
Ill (e.g. with fever, bug)				Touch/tactile stimulation			
During the night				During the evening			
At work				At home (main residence)			
Stress				Too cold or too hot			
Alone				Flickering light			
Thirsty or hungry				At day activities/day centre			
Tired or drowsy				Toward the end of an activity/task			
Walking or other exercise				Hyperventilating/breathing abnormally			
Frustrated				TV or computer games			
In crowded room				Away on holiday			
Sudden change in posture				Sudden noise			
When waking up after sleep				Pain			
Alert				Just after an activity/task has finished			
With a stranger				Bored			
During the afternoon				During the morning			
Surprised/startled				Sitting still			
Being criticised/reprimanded				Doing nothing			
Engaged in work or education activity				Sad			
Sudden change in light levels				With friends or family			
Waiting for an activity/task to begin				Sleep deprived			
Visiting relative or friend's house				Happy			
Frightened				During sleep			
When falling asleep				Anxious			
Washing/dressing				Reading			

Q25. (a) Is there anything **not included in the two lists above** that **increases** the likelihood of seizures of this type in the person you care for? If so please write this here:……………………………………………………………………….

(b) Is there anything **not included in the two lists above** that **decreases** the likelihood of seizures of this type in the person you care for? If so please write this here:……………………………………………………………………….

Q26. What has the doctor treating his/her epilepsy said about this seizure type? Please tick one:

The doctor has said this seizure type is epileptic ☐
The doctor has said s/he is uncertain about whether this seizure type is epileptic ☐
The doctor has said this seizure type is not epileptic ☐
None of the above ☐

Q27. How often does s/he have a seizure **of this type**? Answer this by thinking back over the last year. Please tick 1:

Less often than once a month ☐– if so how many seizures over the past year?……….
About once a month ☐
More often than once a month, but less often than once a week ☐
About once a week ☐
More often than once a week, but less often than once a day ☐
About once a day ☐
More often than once a day ☐ - if so how many per day?…………

--- If you listed more than three seizure types in question 14, please continue to part E. If not, go to part F ---

Part E: Seizure type 4

For the seizure type you described as **'Seizure type 4'** in Question 14, please answer the following questions.

Q28. Some situations are listed below. Please go through **both** lists and tick one column for each situation, to say **whether the situation is associated with** a **decreased** or **increased chance** of the seizures you described as '**Seizure type 4**', in the person you care for. If the situation is not related to whether seizures are likely to occur, please tick 'no association'. If a situation is not applicable to the person you care for, leave it blank and move on to the next one.

**Situation**	Seizures **less likely**	No association	Seizures **more likely**
Example		√	
Outdoors			
Excitement			
Relaxed			
In noisy environment			
Ill (e.g. with fever, bug)			
During the night			
At work			
Stress			
Alone			
Thirsty or hungry			
Tired or drowsy			
Walking or other exercise			
Frustrated			
In crowded room			
Sudden change in posture			
When waking up after sleep			
Alert			
With a stranger			
During the afternoon			
Surprised/startled			
Being criticised/reprimanded			
Engaged in work or education activity			
Sudden change in light levels			
Waiting for an activity/task to begin			
Visiting relative or friend's house			
Frightened			
When falling asleep			
Washing/dressing			

**Situation**	Seizures **less likely**	No association	Seizures **more likely**
Angry			
Menstruating			
Winding down after stress			
Engaged in leisure activity			
Constipated			
Touch/tactile stimulation			
During the evening			
At home (main residence)			
Too cold or too hot			
Flickering light			
At day activities/day centre			
Toward the end of an activity/task			
Hyperventilating/breathing abnormally			
TV or computer games			
Away on holiday			
Sudden noise			
Pain			
Just after an activity/task has finished			
Bored			
During the morning			
Sitting still			
Doing nothing			
Sad			
With friends or family			
Sleep deprived			
Happy			
During sleep			
Anxious			
Reading			

Q29. (a) Is there anything **not included in the two lists above** that **increases** the likelihood of seizures of this type in the person you care for? If so please write this here:...

(b) Is there anything **not included in the two lists above** that **decreases** the likelihood of seizures of this type in the person you care for? If so please write this here:...

Q30. What has the doctor treating his/her epilepsy said about this seizure type? Please tick one:

The doctor has said this seizure type is epileptic ☐

The doctor has said s/he is uncertain about whether this seizure type is epileptic ☐

The doctor has said this seizure type is not epileptic ☐

None of the above ☐

Q31. How often does s/he have a seizure **of this type**? Answer this by thinking back over the last year. Please tick 1:

Less often than once a month ☐– if so how many seizures over the past year?..........

About once a month ☐

More often than once a month, but less often than once a week ☐

About once a week ☐

More often than once a week, but less often than once a day ☐

About once a day ☐

More often than once a day ☐ - if so how many per day?............

--- Please continue to part F ---

Part F: Thank you

Thank you for completing this questionnaire. If you would like to add any further comments please use the space below. Then please return this questionnaire with one copy of the signed consent form, in the FREEPOST envelope provided (no stamp is required).
